# Supplementary material for: Genetic structure of Trifolium pratense populations in a cityscape
Source: PeerJ. 2023 Sep 5;11:e15927. doi: 10.7717/peerj.15927 (PMC10487591; doi:10.7717/peerj.15927)
Supplement: Supplemental Information 2 [file peerj-11-15927-s002.docx]

**Appendix**

**Table S1.** ISSR primers used in this study

| Primer (UBC) name | Primer Sequence | Annealing temperature (T ° C) | Polymorphic bands | |
| --- | --- | --- | --- | --- |
|  |  |  | Number | Size range (bp) |
| 807 | (AG)_8_ T | 50^°^C | 10 | 300 – 900 bp |
| 812 | (GA)_8_ A | 50^°^C | 16 | 200 – 800 bp |
| 825 | (AC)_8_ T | 50^°^C | 15 | 300 – 900 bp |
| 826 | (AC)_8_ G | 52^°^C | 15 | 300 – 900 bp |
| 834 | (AG)_8_ YT | 52^°^C | 5 | 100 – 700 bp |
| 842 | (CT)_8_  YG | 54^°^C | 15 | 150 – 600 bp |
| 846 | (CA)_8_ RT | 54^°^C | 9 | 200 – 850 pb |
| 848 | (CA)_8_ RG | 54^°^C | 10 | 300 – 900 bp |

**Table S2.** Pearson rank correlation coefficient between different measures of within population diversity. All coefficients are significant at p < 0.001.

|  | Na^1)^ | Ne^2)^ | H^3)^ | I^4)^ | Pl^5)^ | Pl [%]^6)^ |
| --- | --- | --- | --- | --- | --- | --- |
| Na^1)^ |  | 0.902 | 0.967 | 0.979 | 1.000 | 1.000 |
| Ne^2)^ | 0.902 |  | 0.971 | 0.957 | 0.902 | 0.902 |
| H^3)^ | 0.967 | 0.971 |  | 0.998 | 0.967 | 0.967 |
| I^4)^ | 0.979 | 0.957 | 0.998 |  | 0.979 | 0.979 |
| Pl^5)^ | 1.000 | 0.902 | 0.967 | 0.979 |  | 1.000 |
| Pl [%]^6)^ | 1.000 | 0.902 | 0.967 | 0.979 | 1.000 |  |

1. Na = Observed number of alleles
2. Ne = Effective number of alleles (Kimura and Crow (1964))
3. H = Nei’s (1973) gene diversity
4. I = Shannon’s information index (Lewontin (1972))
5. Pl = number of polymorphic loci
6. Pl [%] = percentage of polymorphic loci (Pl)
7. Pb = number of private loci

**Figure S1.** Values of delta K for different numbers of subpopulations (K) computed for populations *Trifolium pratense* based on Evanno’s delta K estimation (Evanno et al. 2005)*.*

*
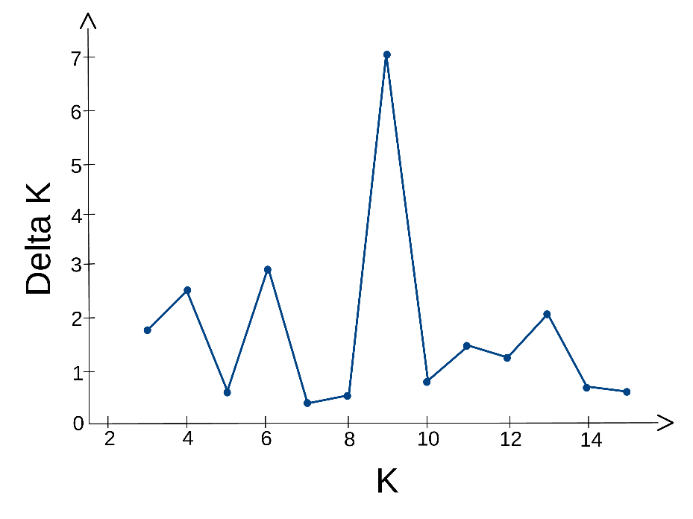
*

**Figure S2.** Population genetic structure determined by the program STRUCTURE (Pritchard et al. 2000) for different numbers of clusters (K). The vertical columns represent individual plants. The colours correspond to the assignment of each plant to a genetic cluster. Plants and populations that show multiple colours provide evidence for admixture. The results for 9 clusters are highlighted, based on values of delta K (see Figure S1). Above and below the graph, the number of the particular population, consistent with Figure 1 and Table 1, is also shown, as well the membership for a particular cluster (lowercase letter, under the population number).


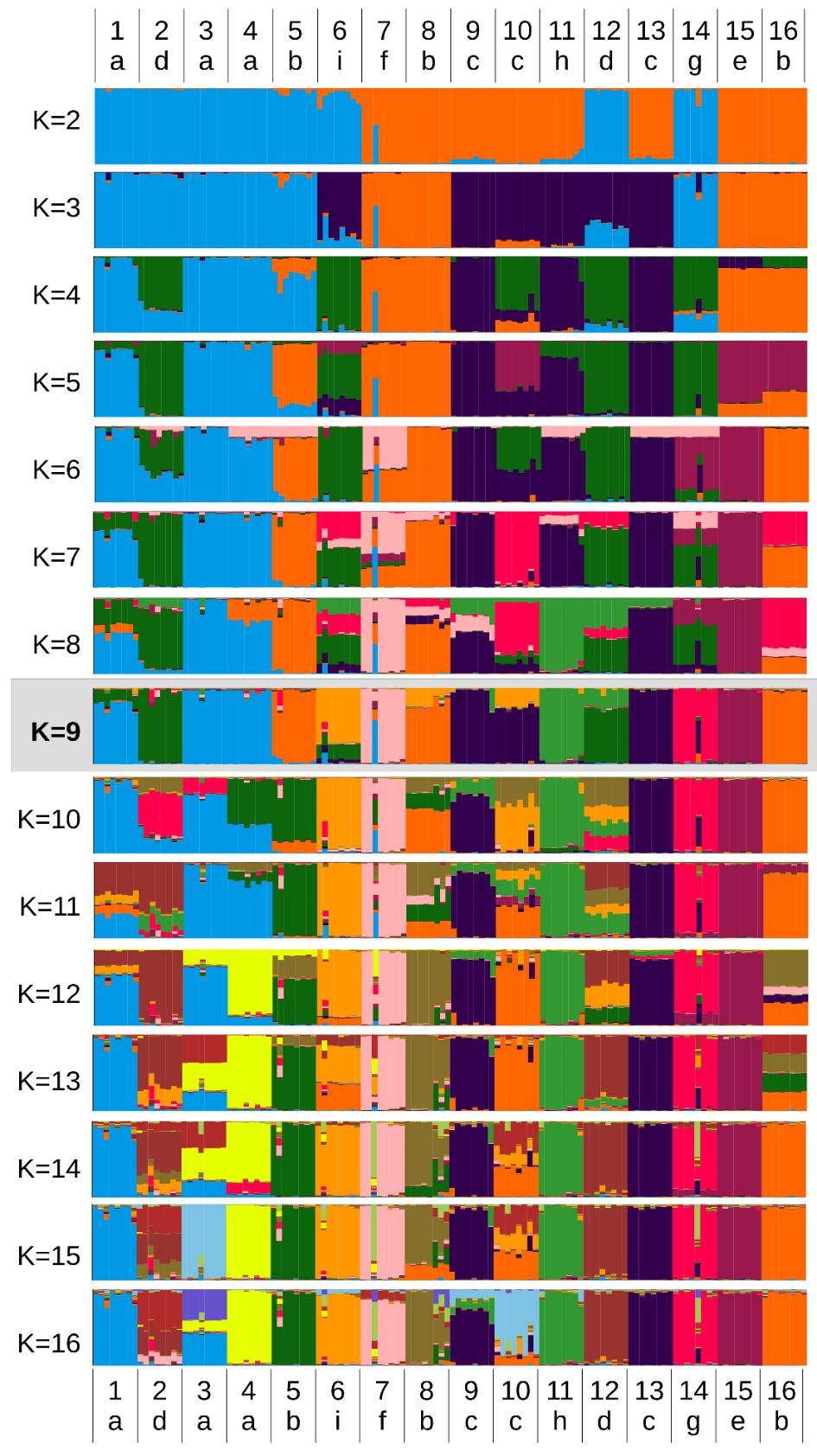


**Least cost path analysis**

The layers downloaded in ‘shp’ format and land cover data from original BDOT10k, were merged, and information regarding land cover type was transformed into resistance values ranging from 1 to 100:

- no resistance: 1 (urban grasslands)
- low: 5 (forests, parks, allotments)
- moderate: 20 (rivers, disperse buildings, arable grounds)
- high: 100 (built-up areas, communications and remaining).

Detailed information on values of resistance for particular land cover types in the original BDOT10k Level 1 and Level 2 applied here is presented in Table S3. The resistance values for resistance surface were parametrized based on expert opinion. We focused on pollen movement by pollinators, particularly bumblebees, excluding seed movement related to large herbivores, which were less likely to be present in a city. The information regarding habitat preferences of the pollinators in Wrocław were taken from Michołap et al. (2017) and Sikora and Michołap (2018). Quantification of resistance surface is problematic (for reviews, see Zeller et al. 2012, Peterman et al. 2019), and we optimized resistance surfaces based on genetic data (Peterman 2018). However, since there was likely a lag effect between the recently observed landscape configuration and the genetic structure of surveyed populations (Epps & Keyghobadi 2015), we did not optimise the resistance values by comparing them with genetic data.

Next, the merged shape file with resistance values was transformed to ‘geotiff’ file with 5 × 5 m resolution. The ‘geotiff’ file was opened in the ‘gdistance’ package in R environment and transformed to ‘TransitionLayer’ object and used for LCP analysis in the ‘leastcostpath’ package. For transformation in the ‘gdistance’ package, we used the following settings:

‘1/resist’ – input raster (resist) converted from resistance values to transition (1/resist)

‘mean’ – transition function

‘8’ – number of connected cells

Since the LCP calculations using ‘from each to each’ algorithm are computational very demanding (White & Barber 2012), we simplified them by providing geographical coordinates of population centroids only. Then, the LCP length matrix was multiplied to size 128 × 128 (for each individual) using the value of 10 m as the LCP length between individuals within a population. The values corresponded to the minimal distance between individuals sampled in the field, and to the resistance value 1 for migration within an urban grassland path. It only slightly influenced the results because the shortest LCP between 2 populations was about 1 km, which was larger than interpatch LCPs by 2 orders of magnitude.

**Table S3.** Resistance values assigned to particular land cover types distinguished in the BDOT10k database.

| **Land cover types** | | **resistance values** |
| --- | --- | --- |
| **Class 1** | **Class 2** |  |
| water surface | salt water | 20 |
|  | runing water | 20 |
|  | stagnant water | 20 |
| build-up areas | multi-family housing | 100 |
|  | single-family housing | 20 |
|  | industrial and storage buildings | 100 |
|  | commercial and service development | 100 |
|  | remaining build-up areas | 100 |
| woodlands | forests | 5 |
|  | woodlots | 5 |
|  | tree shelterbelts | 5 |
|  | *Pinus mugo* shrubland | 5 |
|  | shrubland | 5 |
| plantations | allotments | 5 |
|  | plantations | 20 |
|  | orchards | 20 |
|  | forest nursery | 20 |
|  | ornamental plant nursery | 20 |
| grasslands and arable lands | grasslands | 1 |
|  | arable lands | 20 |
| areas under communication routes | vehiculars traffic roads | 100 |
|  | railroads | 100 |
|  | vehiculars traffic roads and railroads | 100 |
|  | airstrips and airport roads | 100 |
| unused grounds | scree, or rock rubble | 100 |
|  | rocky areas | 100 |
|  | sandy areas | 100 |
|  | pozostały grunt nieużytkowany | 100 |
| places | town squares, public squares | 100 |
| landfill areas | municipal waste storage area | 100 |
|  | industrial waste storage area | 100 |
| excavations and dumping grounds | excavations | 100 |
|  | heaps | 100 |
| remaining undeveloped land | area under technical devices or buildings | 100 |
|  | industrial and storage area | 100 |

**Figure S3**. Map of least cost paths (LCPs) on the background of resistance surface. For map simplification, only the locations of population centroids are shown. The OpenStreet map (<https://www.openstreetmap.org> (C) OpenStreetMap contributors, CC BY-SA 2.0) is used in the background.


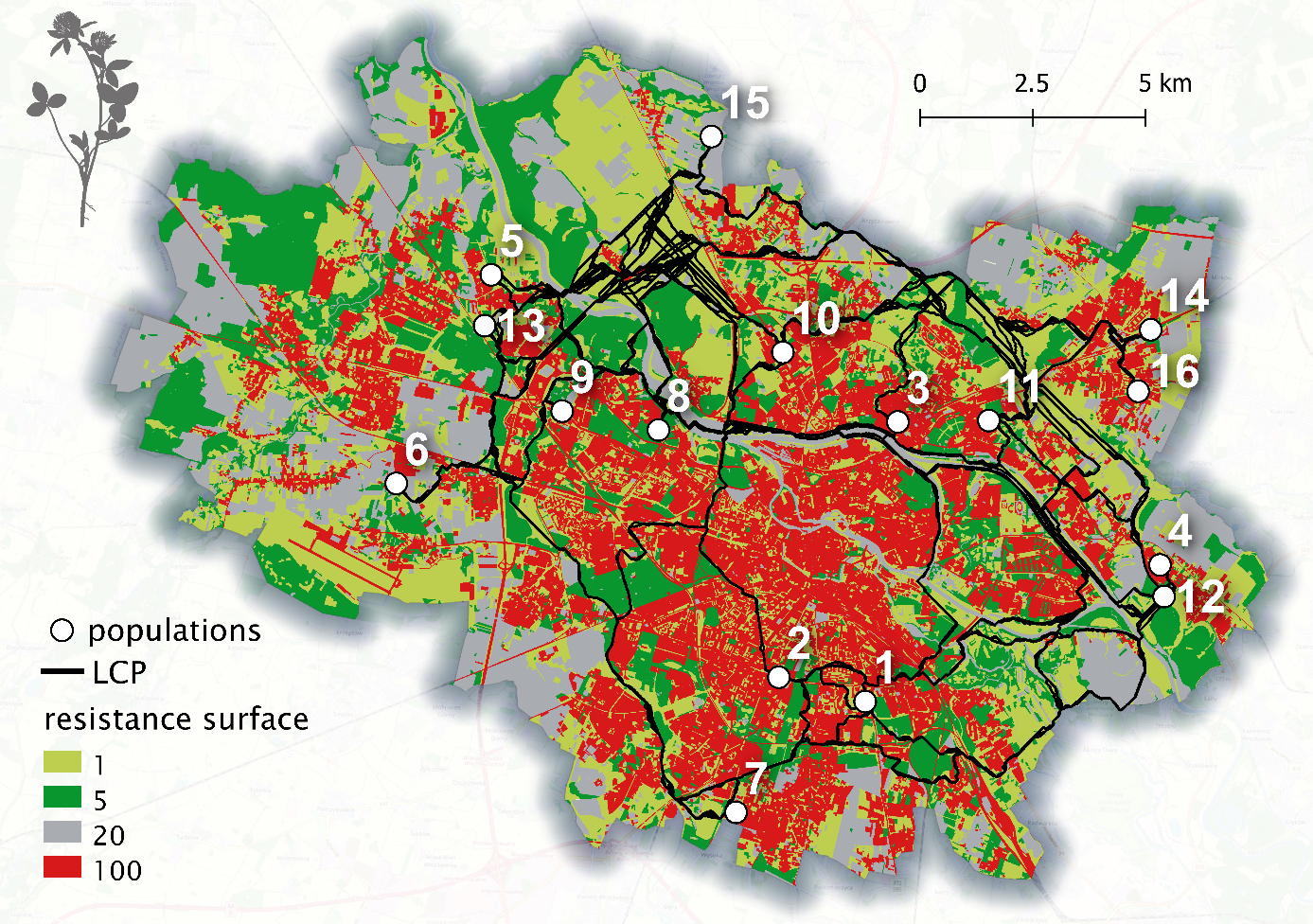


**Table S4.** Spearman rank correlations coefficient (r) and its probability (p) between Nei’s genetic diversity in each population and structural connectivity measures for different distance thresholds. The details of integral index of connectivity (IIC) and its interpretation can be found in Saura and Torné (2009), Saura and Rubio (2010), and Baranyi et al. (2011), while details of calculations for Wrocław urban grasslands are available in Mollashahi et al. (2020).

| Distance threshold [m] | measures | r | p |
| --- | --- | --- | --- |
| 2 | dIIC | -0.009 | 0.973 |
| 2 | *intra* | 0.421 | 0.105 |
| 2 | *flux* | -0.421 | 0.105 |
| 2 | *connectivity* | 0.000 | 0.000 |
| 20 | dIIC | 0.040 | 0.883 |
| 20 | *intra* | -0.004 | 0.987 |
| 20 | *flux* | -0.205 | 0.447 |
| 20 | *connectivity* | 0.071 | 0.793 |
| 44 | dIIC | 0.005 | 0.985 |
| 44 | intra | -0.236 | 0.380 |
| 44 | flux | 0.203 | 0.450 |
| 44 | connectivity | 0.044 | 0.871 |
| 100 | dIIC | -0.066 | 0.807 |
| 100 | *intra* | -0.024 | 0.931 |
| 100 | *flux* | 0.034 | 0.901 |
| 100 | *connectivity* | -0.013 | 0.961 |
| 1000 | dIIC | -0.071 | 0.795 |
| 1000 | *intra* | -0.109 | 0.688 |
| 1000 | *flux* | -0.063 | 0.816 |
| 1000 | *connectivity* | 0.089 | 0.743 |

dIIC – delta of Integral Index of Connectivity

*intra* – fraction of dIIC values related to connectivity within the patch

*flux* – fraction of dIIC values which indicates whether the patch is directly connected to another

*connectivity* – fraction of dIIC values which indicates whether a node acts as a stepping-stone and contributes to the connection between other nodes

**Urbanisation gradient**

The BDO10k map was also used to map the impervious surface. Following Bartlewicz et al. (2015), we assumed that the surface of buildings and road corridors, were impervious surface. In addition, owing to the BDOT10k map characteristics, we also included city squares with sealed surface, and entire build-up areas in the categories ‘industrial and storage buildings’ and ‘commercial and service development’. Next the merged shape files were transformed to ‘geotiff’ file with 5 × 5 m resolution, and the percentage of the urbanised surface within the buffers was calculated. The distribution of impervious surfaces within Wrocław city is presented on Figure S4.

**Figure S4.** The distribution of impervious surfaces within Wrocław city. The OpenStreet map (<https://www.openstreetmap.org> (C) OpenStreetMap contributors, CC BY-SA 2.0) is used in the background.


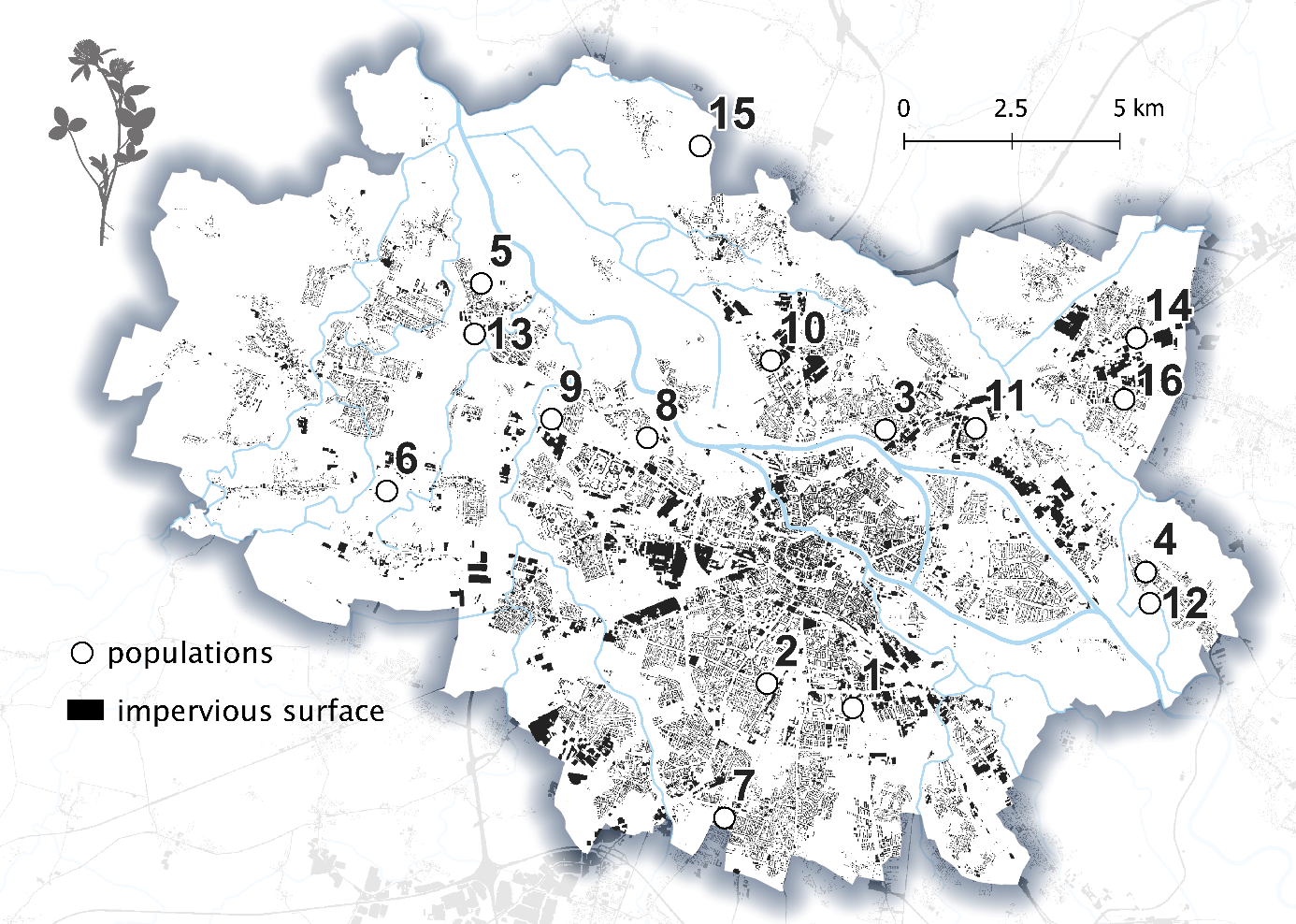


**Table S5.** Spearman rank correlations coefficient (r) and its probability (p) between Nei’s genetic diversity in each population and urbanization level (urb%) in buffers with radii of 0.5, 1, and 2 km.

| urbanization level | r | p |
| --- | --- | --- |
| urb%_0.5km | 0.225 | 0.401 |
| urb%_1km | 0.219 | 0.414 |
| urb%_2km | 0.401 | 0.124 |

**References**

Baranyi, G., Saura, S., Podani, J., Jordán, F. (2011). Contribution of habitat patches to network connectivity: Redundancy and uniqueness of topological indices. Ecological Indicators, 11(5), 1301-1310.

Bartlewicz, J., Vandepitte, K., Jacquemyn, H., & Honnay, O. (2015). Population genetic diversity of the clonal self-incompatible herbaceous plant Linaria vulgaris along an urbanization gradient. Biological Journal of the Linnean Society, 116(3), 603-613.

Epps, C. W., & Keyghobadi, N. (2015). Landscape genetics in a changing world: disentangling historical and contemporary influences and inferring change. Molecular ecology, 24(24), 6021-6040.

Evanno, G., Regnaut, S., & Goudet, J. (2005). Detecting the number of cluster of individuals using the software Structure: A simulation study. Molecular Ecology, 14, 2611– 2620. https://doi.org/10.1111/j.1365-294X.2005.02553.x

Fortel, L., Henry, M., Guilbaud, L., Guirao, A. L., Kuhlmann, M., Mouret, H., ... & Vaissière, B. E. (2014). Decreasing abundance, increasing diversity and changing structure of the wild bee community (Hymenoptera: Anthophila) along an urbanization gradient. PloS one, 9(8), e104679.

Michołap, P., Sikora, A., Kelm, M., & Sikora, M. (2017). Variability of bumblebee communities (Apidae, Bombini) in urban green areas. Urban Ecosystems, 20, 1339-1345.

Mollashahi, H., Szymura, M., & Szymura, T. H. (2020). Connectivity assessment and prioritization of urban grasslands as a helpful tool for effective management of urban ecosystem services. PLoS One, 15(12), e0244452.

Peterman, W. E. (2018). ResistanceGA: An R package for the optimization of resistance surfaces using genetic algorithms. Methods in Ecology and Evolution, 9(6), 1638-1647.

Peterman, W. E., Winiarski, K. J., Moore, C. E., Carvalho, C. D. S., Gilbert, A. L., & Spear, S. F. (2019). A comparison of popular approaches to optimize landscape resistance surfaces. Landscape Ecology, 34, 2197-2208.

Saura S., Torné J., (2009) Conefor sensinode 22: a software package for quantifying the importance of habitat patches for landscape connectivity. Environ Model Softw 24: 135–139.

Saura S., Rubio L., (2010) A common currency for the different ways in which patches and links can contribute to habitat availability and connectivity in the landscape. Ecography 33(3): 523-537.

Sikora A., Michołap P., 2018. Pszczoły w mieście. Trzmiele Wrocławia. Stowarzyszenie Natura i Człowiek. Wrocław 2018

White, D. A., & Barber, S. B. (2012). Geospatial modeling of pedestrian transportation networks: a case study from precolumbian Oaxaca, Mexico. Journal of archaeological science, 39(8), 2684-2696.

Zeller, K. A., McGarigal, K., & Whiteley, A. R. (2012). Estimating landscape resistance to movement: a review. Landscape ecology, 27, 777-797.
